# The requirement of SUMO2/3 for SENP2 mediated extraembryonic and embryonic development

**DOI:** 10.1002/dvdy.125

**Published:** 2019-11-01

**Authors:** H‐M Ivy Yu, Trunee Hsu, Eri O Maruyama, Wulf Paschen, Wei Yang, Wei Hsu

**Affiliations:** ^1^ Center for Oral Biology University of Rochester Medical Center Rochester New York; ^2^ Pittsford Mendon High School Pittsford New York; ^3^ Department of Anesthesiology Duke University Durham North Carolina; ^4^ Department of Biomedical Genetics University of Rochester Medical Center Rochester New York; ^5^ Stem Cell and Regenerative Medicine Institute, University of Rochester Medical Center Rochester New York

**Keywords:** SUMO, protease, SENP, SUMOylation, placental insufficiency, cardiovascular deformity, mouse genetics

## Abstract

Small ubiquitin‐related modifier (SUMO)‐specific protease 2 (SENP2) is essential for the development of healthy placenta. The loss of SENP2 causes severe placental deficiencies and leads to embryonic death that is associated with heart and brain deformities. However, tissue‐specific disruption of SENP2 demonstrates its dispensable role in embryogenesis and the embryonic defects are secondary to placental insufficiency. SENP2 regulates SUMO1 modification of Mdm2, which controls p53 activities critical for trophoblast cell proliferation and differentiation. Here we use genetic analyses to examine the involvement of SUMO2 and SUMO3 for SENP2‐mediated placentation. The results indicate that hyper‐SUMOylation caused by SENP2 deficiency can be compensated by reducing the level of SUMO modifiers. The placental deficiencies caused by the loss of SENP2 can be alleviated by the inactivation of gene encoding SUMO2 or SUMO3. Our findings demonstrate that SENP2 genetically interacts with SUMO2 and SUMO3 pivotal for the development of three major trophoblast layers. The alleviation of placental defects in the SENP2 knockouts further leads to the proper formation of the heart structures, including atrioventricular cushion and myocardium. SUMO2 and SUMO3 modifications regulate placentation and organogenesis mediated by SENP2.

## Key Findings

- Genetic analyses reveal that hyper sumoylation caused by SENP2 deficiency can be compensated by reducing the level of SUMO modifiers. The placental deficiencies caused by the loss of SENP2 can be alleviated by the inactivation of gene encoding SUMO2 or SUMO3. SENP2 genetically interacts with SUMO2 and SUMO3 pivotal for the development of three major trophoblast layers. The alleviation of placental defects in the SENP2 knockouts further leads to the proper formation of the heart structures, including atrioventricular cushion and myocardium. Protein modification by SUMO2 and SUMO3 is essential for SENP2‐mediated placentation and organogenesis.

## 1 INTRODUCTION

Covalent conjugation of proteins by small ubiquitin‐related modifier (SUMO) is a reversible and evolutionary conserved process.^1^, ^2^ SUMO modification can modulate a variety of cellular functions, including protein trafficking, cell cycle and cell survival/death.^3^, ^4^, ^5^, ^6^, ^7^ SUMO has been shown to alter protein function, activity, interaction, and subcellular distribution. The transfer of SUMO polypeptides to their targets is called “SUMOylation”, catalyzed by E3 ligases.^1^, ^8^ The reversed “deSUMOylation” process which removes SUMO is mediated by SUMO‐specific proteases.^9^, ^10^ The hallmark of these proteases is a highly conserved SENP domain located at the carboxyl terminus. They catalyze deSUMOylation in various physiological systems, and genetic analysis has recently begun to unfold their importance in mammalian development and disease.^11^, ^12^, ^13^, ^14^, ^15^

Genetic inactivation of SUMO‐specific protease 2 (*SENP2*) in mice reveals its irreplaceable function for the development of trophoblast stem cell niches and lineages during placentation.^11^ In addition to placental defects, the global knockout of SENP2 also causes embryonic lethality and the mutants exhibit abnormalities in the brain and heart.^12^, ^13^ The placental insufficiency greatly complicates the analysis of these phenotypes as they arise at specific stages when embryogenesis becomes highly dependent on placental function.^11^ Further analyses using the conditional deletion approach revealed that the brain and heart abnormalities of SENP2 global knockout are not primary, but secondary defects due to placental deficiencies.^12^, ^14^ With a healthy placenta containing intact SENP2, embryos with epiblast‐specific ablation of SENP2 did not exhibit any brain and heart defects, demonstrating the contribution of placental insufficiency to the observed embryonic deformities.

The loss of SENP2 resulted in its substrates maintained at the sumoylated state. Their hyper‐SUMOylation is likely the cause of the extraembryonic deformities. We previously identified that SENP2 mediates SUMO1 modification of Mdm2/p53 signaling, contributing to the cell cycle regulation of trophoblast stem cell proliferation and differentiation.^11^ There are four confirmed SUMO polypeptides.^1^, ^6^, ^8^, ^16^ However, it remains to be determined if SENP2 also modulates other SUMO regulatory pathways, for example, SUMO2 and SUMO3, essential for placentation. To determine the role of SUMO2 and SUMO3 in SENP2‐mediated extraembryonic development, we performed genetic tests for SENP2, SUMO2, and SUMO3. By reducing the amount of SUMO2/3, we tested if placental defects caused by hyper‐SUMOylation could be alleviated in the SENP2 null. The results of our genetic analysis clearly demonstrated the requirement of SUMO2 and SUMO3 for SENP2‐mediated placentation that is essential for the development of the healthy embryo.

## 2 RESULTS

### 2.1 Genetic interaction of SENP2 with SUMO2 and SUMO3

**Figure 1:**
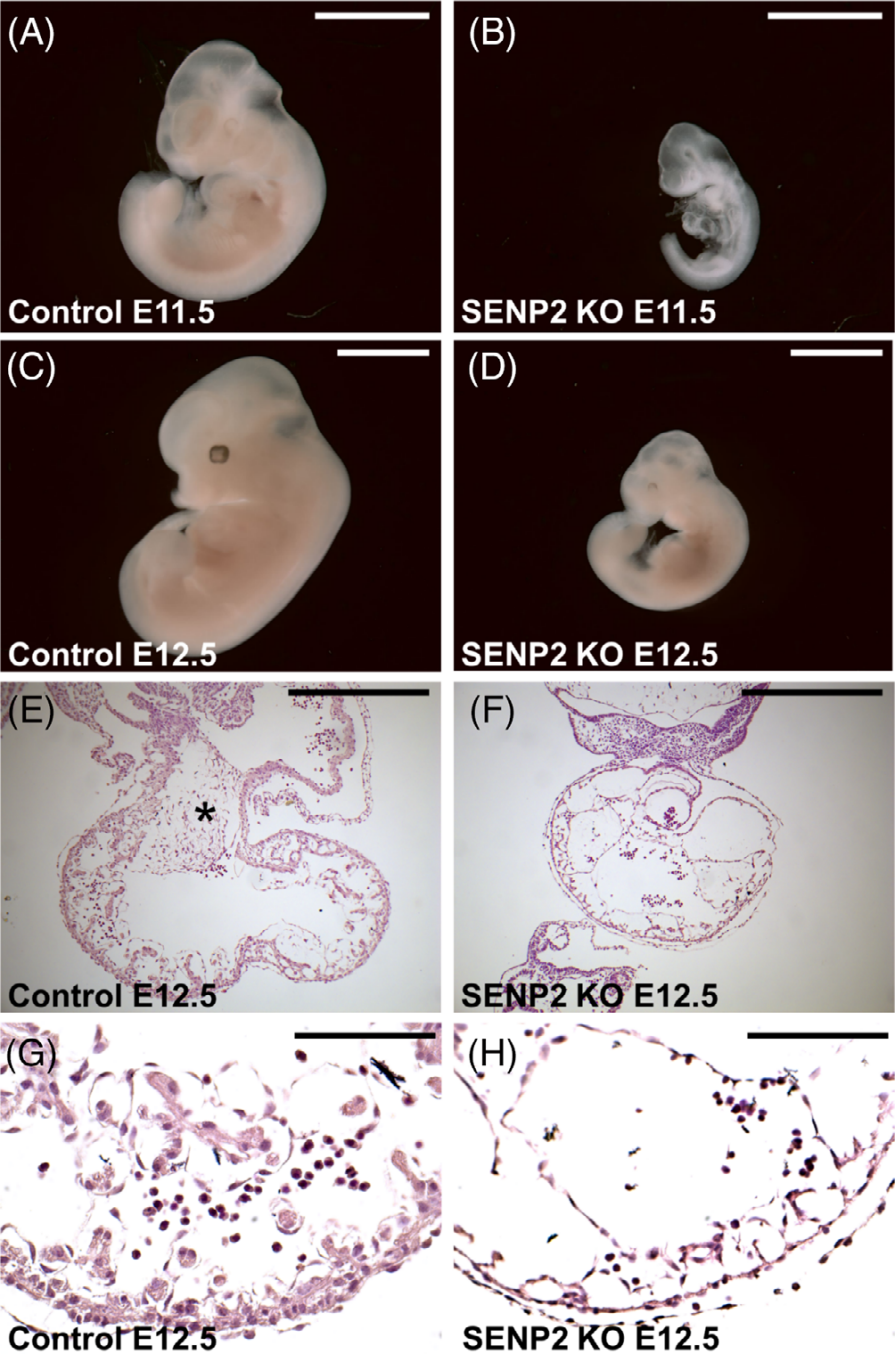
Secondary defects in heart formation caused by placental deficiencies in the SENP2 knockout. Gross morphological evaluation of the wild type (A, C) and SENP2−/− (B, D), embryos at E11.5 (A‐B) and 12.5 (C‐D). Histology shows the atrioventricular (AV) cushion (E‐F; asterisks) and myocardium (G‐H) defective in the SENP2 knockout. Scale bars, 3 mm (A‐D); 100 μm (E‐H). SENP2, SUMO‐specific protease 2

SENP2 is required for SUMO1‐mediated trophoblast development and the ablation of SENP2 causes placental deficiencies leading to embryonic death at mid‐gestation where the embryo begins to reply on maternal supplies upon allantoic fusion.^11^ Intercross of SENP2+/− mice resulted in the homozygous embryos, which appear underdeveloped after E9.5 and most of them did not survive. Only a few SENP2 nulls could be recovered after E11.5 but were significantly underdeveloped (Figure 1A‐D) and exhibited heart deformities including pericardial effusion, missing atrioventricular (AV) cushion (Figure 1E,F), and myocardial thinning (Figure 1G,H). As we demonstrated previously using conditional knockout mouse models, these were secondary defects caused by placental insufficiencies.^14^

**Figure 2:**
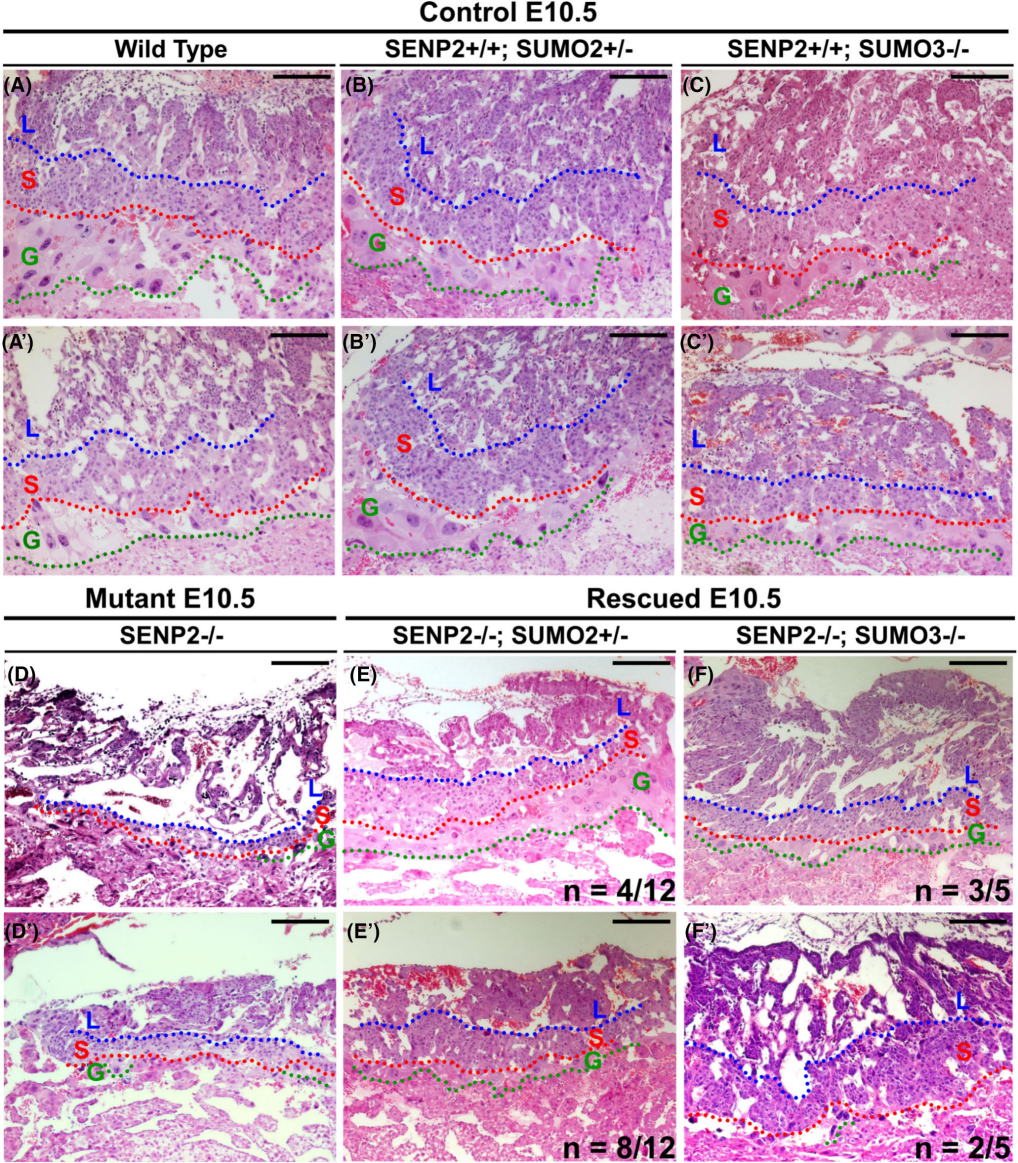
SENP2 genetically interacts with SUMO2 and SUMO2 during the formation of three major trophoblast layers. Histology examines the placentas of SENP2+/+ (A, A′) and SENP2+/+; SUMO2+/− (B, B′), SENP2+/+; SUMO3−/− (C, C′), SENP2−/− (D, D′), SENP2−/−; SUMO2+/− (E, E′) and SENP2−/−; SUMO3−/− (F, F′) in transverse sections at E10.5. Labyrinth (L), spongiotrophoblast (S) and trophoblast giant cell (G) layers were defined by blue, red and green broken lines, respectively. Scale bars, 200 μm (A‐F, A′‐F′). SENP, SUMO‐specific protease; SUMO, small ubiquitin‐related modifier

SUMO2 and SUMO3 show an extremely high degree of similarity (approximately 95% identical). Therefore, we used a genetic approach to test if SUMO2 and SUMO3 also modulate placental development mediated by SENP2. To perform genetic tests, mice carrying the deletion of SENP2 were crossed with those carrying SUMO2 and SUMO3 mutation to obtain the double mutants. At E10.5, three major trophoblast layers — the labyrinth, spongiotrophoblast, and TGC (trophoblast giant cell) in SUMO2+/− and SUMO3−/− placentas are comparable to the wild type (Figure 2A‐C and A′‐C′; Control n = 12). Similar to our prior reports,^11^, ^14^ the loss of SENP2 impaired placental development (Figure 2). The SENP2−/− placentas were smaller and paler than the controls. The development of all three trophoblast layers that cannot be distinguished by histological analysis (Figure 2D,D′; n = 2). The TGC layer, most severely affected by the loss of SENP2, is almost completely missing. To test if removal of SUMO2 or SUMO3 modifiers could reduce the hyper‐sumoylated effects in the SENP2−/− placenta, we performed genetic tests. The results showed three trophoblast layers clearly formed in the SENP2−/−; SUMO2+/− (Figure 2E; n = 4/12) and SENP2−/−; SUMO3−/− (Figure 2F; n = 3/5) placentas. Removing one allele of SUMO2 seemed to have more alleviative effects than removing two alleles of SUMO3 (Figure 2E‐F). Unfortunately, we could not test the removal of two alleles of SUMO2 due to embryonic lethality.^17^ In some cases, the alleviation is less effective (Figure 2E′; n = 8/12 and Figure 2F′; n = 2/5). The results clearly demonstrated that SENP2 genetically interacts with SUMO2 and SUMO3. The SENP2‐dependent SUMO2 and SUMO3 modifications are required for placental development.

### 2.2 Reversal of placental deficiencies alleviates embryonic heart defects in the SENP2 nulls

**Figure 3:**
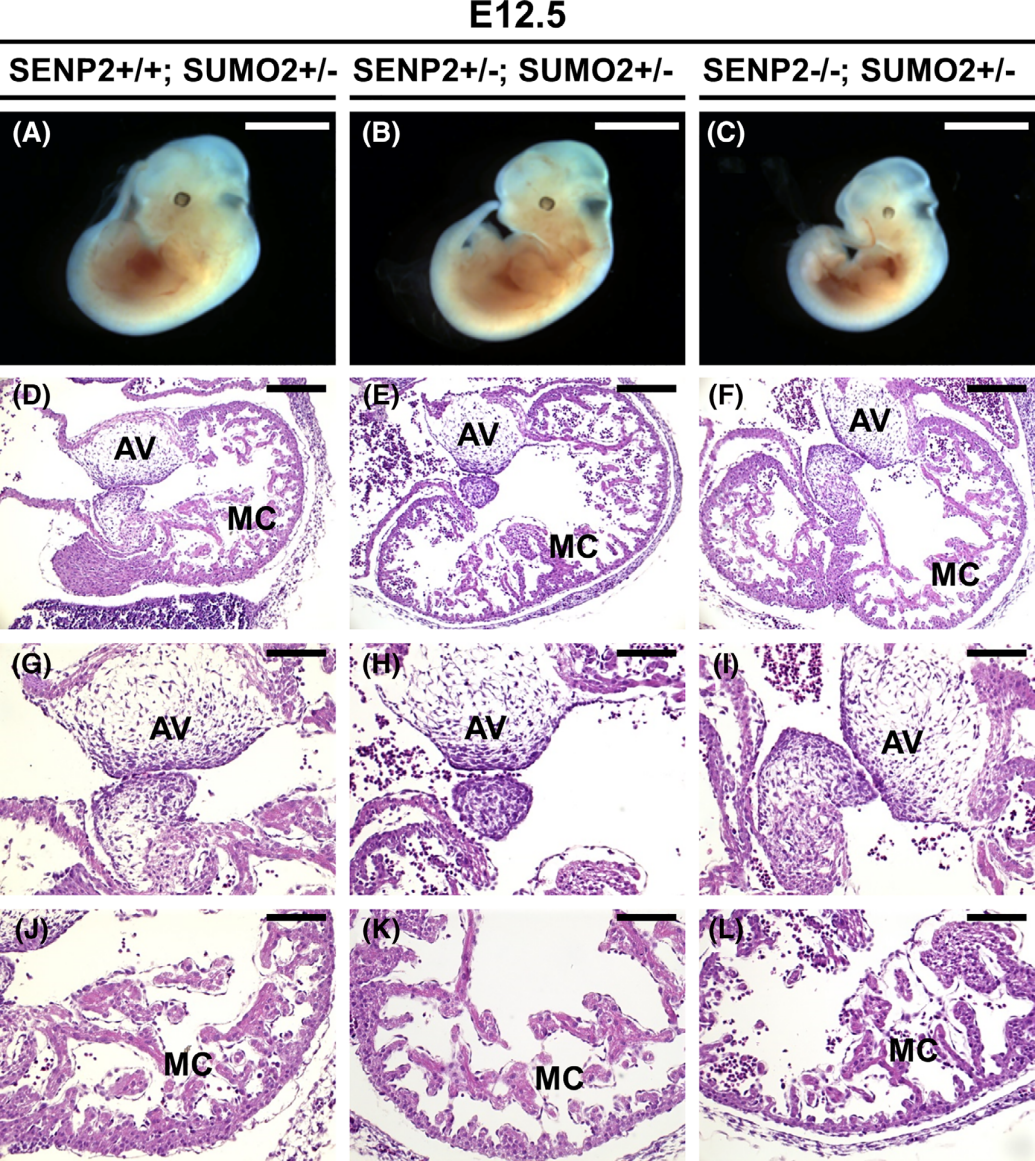
Alleviation of heart deformities caused by SENP2 deficiency. (A‐C) Gross morphological evaluation of the SENP2+/+; SUMO2+/− (A), SENP2+/−; SUMO2+/− (B) and SENP2−/−; SUMO2+/− (C) embryos at E12.5. Histology shows embryo homozygous for SENP2 and heterozygous for SUMO2 (F, I, L) exhibiting the proper formation of the atrioventricular (AV) cushion (D‐I) and myocardium (MC; D‐F, J‐L) comparable to the control (D‐E, G‐H, J‐K). Scale bars, 4 mm (A‐C); 200 μm (D‐L). SENP, SUMO‐specific protease; SUMO, small ubiquitin‐related modifier

The alleviation of placental defects seemed to allow the embryo to survive after E12.5. We were able to recover SENP2−/−; SUMO2+/− embryos at E12.5 (Figure 3A‐C). Therefore, we examined if the heart deformities are also alleviated by reducing the level of SUMO2. In the SENP2 nulls, the embryonic heart was affected due to placental insufficiencies.^14^ The mutants cardiac showed marked myocardial thinning and missing of atrioventricular (AV) cushions (Figure 1E‐H). Similar to the controls (genotypes: SENP2+/+; SUMO2+/− and SENP2+/−; SUMO2+/−), AV cushion was able to form and easily identifiable in the SENP2−/−; SUMO2+/− embryo (Figure 3D‐I). The development of the myocardium was also comparable to the controls (Figure 3J‐L).

**Figure 4:**
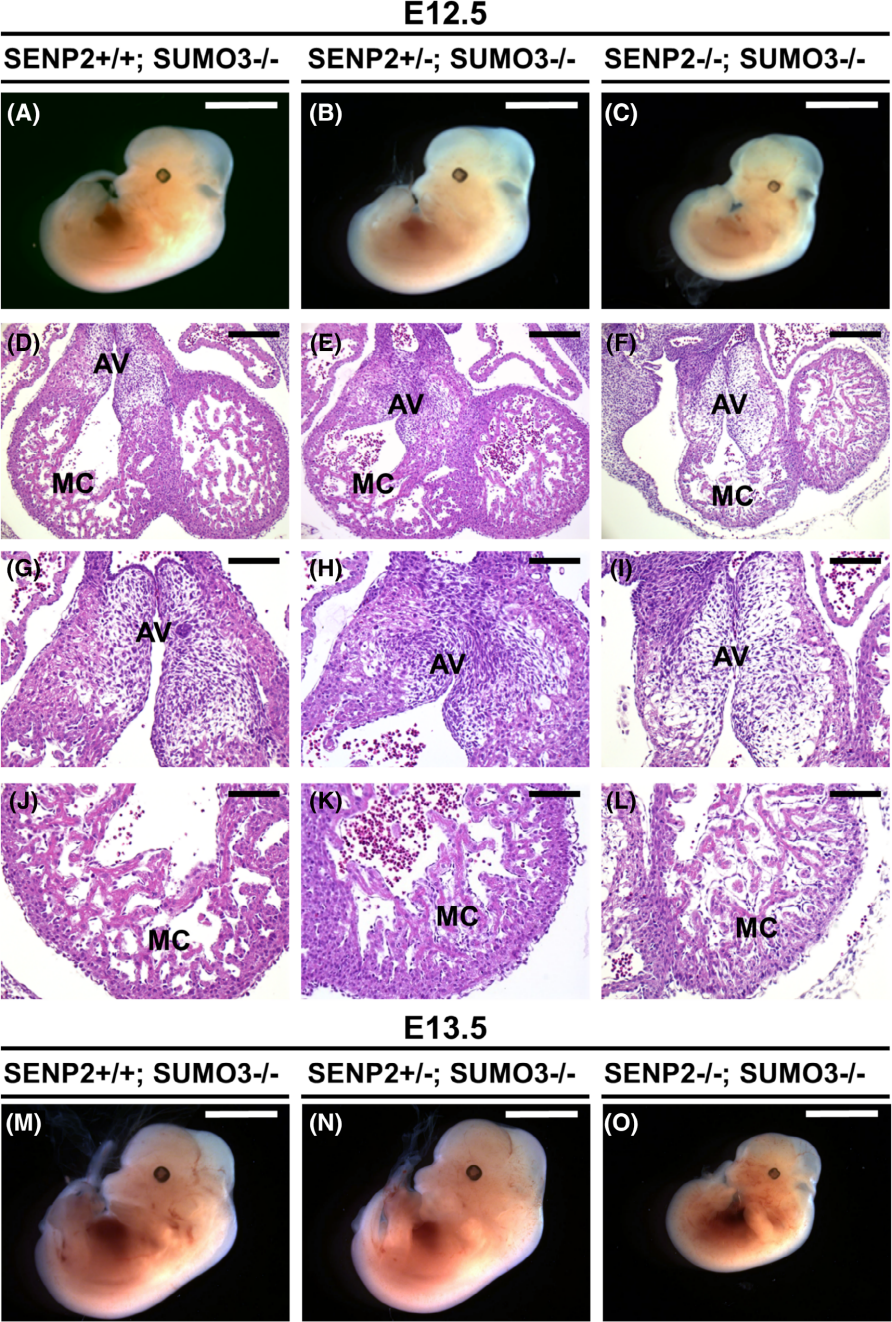
The loss of SUMO3 alleviates heart deformities associated with the SENP2 ablation. (A‐C) Gross morphological evaluation of the SENP2+/+; SUMO3−/− (A, M), SENP2+/−; SUMO3−/− (B, P) and SENP2−/−; SUMO3−/− (C, O) embryos at E12.5 (A‐C) and 13.5 (M‐O). Histology analysis reveals that the SENP2 and SUMO3 double knockout (F, I, L) displays the proper formation of the atrioventricular (AV) cushion (D‐I) and myocardium (MC; D‐F, J‐L) comparable to the control (D‐E, G‐H, J‐K). Scale bars, 4 mm (A‐C, M‐O); 200 μm (D‐F); 100 μm (G‐L). SENP, SUMO‐specific protease; SUMO, small ubiquitin‐related modifier

To test if SUMO3 also modulates SENP2‐dependent embryonic and extraembryonic development, SENP2 nulls were crossed into the SUMO3 homozygous background. We successfully obtained the double knockout of E12.5 and E13.5 embryos (Figure 4A‐C, M‐O), subsequently analyzed by histology for heart development. The removal of SUMO3 was able to alleviate heart defects found in the SENP2 nulls (Figure 4D‐L). Comparable to the control (genotypes: SENP2+/+; SUMO3−/− and SENP2+/−; SUMO3−/−), the AV cushion and myocardium were able to develop in the SENP2−/−; SUMO3−/− embryo (Figure 4D‐L).

## 3 DISCUSSION

Genetic analyses described in this study clearly demonstrate that SUMO2 and SUMO3 modulate SENP2‐dependent extraembryonic and embryonic development. Placental defects caused by hyper‐SUMOylation in the SENP2 knockouts can be alleviated by the reduction of SUMO modifiers. We succeed to prove the concept that hyper SUMOylation caused by the loss of SUMO proteases can be compensated by decreasing the level of SUMO modifiers. Although SUMO1 regulates the Mdm2/p53 pathway essential for cell cycle progression,^11^, ^18^ the important role of SUMO2 and SUMO3 in trophoblast development remains elusive. SUMO2/3 has not only a much wider expression pattern in the trophoblast lineages and cell types, but also distinct stress responses compared to the SUMO1, implying a functional difference between SUMO1 and SUMO2/3 during placental development.^19^ Our findings support the critical functions of SUMO2 and SUMO3 in placentation. Reducing the level of SUMO2 or SUMO3 successfully alleviates the developmental defects of the trophoblast layers, suggesting their involvement in hyper‐SUMOylation caused by the loss of SENP2. To our knowledge, the current study provides the first genetic evidence indicating the essential function of SUMO2 and SUMO3 modifications in placental development.

It remains possible that the alleviation of extraembryonic and embryonic defects is independent of each other in the double mutants. However, our data show a tight link of heart deformities to placental deficiencies, suggesting these are dependent events. Furthermore, our demonstration of SENP2 dispensable for embryonic development strongly argues against a specific requirement of SENP2 in cardiac tissue during heart development.^14^ The heart deformities associated with global inactivation of *SENP2* are not primary but secondary defects due to placental insufficiency. This is further supported by the current study showing that rescue of placental insufficiencies promotes the proper formation of the AV cushion and myocardium.

While removing one allele of SUMO3 has no effect on the SENP2‐null defects, placental and embryonic defects are alleviated by SUMO2 heterozygosity. Therefore, SUMO2 may play a more important role than SUMO3 in SENP2‐mediated placentation. The genetic analyses also suggest nonredundant functions of SUMO2 and SUMO3 in SENP2‐dependent regulation. Although SUMO2 and SUMO3 are ~95% identical, we do not find synergistic effects when both of them are reduced in the SENP2 nulls. It is possible that SUMO2 and SUMO3 modify distinct substrates critical for placentation. In addition to the SUMO1‐mediated modulation, SUMO2/3 may be critical for the regulation of S phase during cell cycle progression.^20^ SUMOylation may also regulate the hypoxia pathway that has been suggested in preeclampsia.^21^ The importance of SUMO2/3 has also been implicated in chromatin remodeling, DNA damage‐induced apoptosis, oxidative stress and cell differentiation.^22^, ^23^, ^24^, ^25^, ^26^ Although the SENP2 substrates modified by SUMO2/3 remain unknown during placentation, it is possible to reveal their identities using proteomic approaches.^27^, ^28^ Identification of these SUMO2/3 targets promises new insight into SENP2‐mediated regulation during extraembryonic and embryonic development.

## 4 EXPERIMENTAL PROCEDURES

### 4.1 Mouse Strains

The SENP2, SUMO2 and SUMO3 knockout mouse strains and their genotyping methods were reported previously.^11^, ^14^, ^17^ The SENP2 and SUMO2 double mutants (genotype: SENP2−/−; SUMO2+/−), and positive (genotype: SENP2−/−; SUMO2+/+) and negative controls (SENP2+/+; SUMO2+/+, SENP2+/+; SUMO2+/−; SENP2+/−; SUMO2+/−) were obtained by intercross between SENP2+/−; SUMO2+/− male and female mice that are viable and fertile. The SENP2 and SUMO3 double mutants (genotype: SENP2−/−; SUMO3−/−), and positive (genotype: SENP2−/−; SUMO3+/+) and negative controls (SENP2+/+; SUMO3−/−, SENP2+/−; SUMO3−/−) were obtained by crossing SENP2+/−; SUMO3+/− males with SENP2+/−; SUMO3+/− or SENP2+/−; SUMO3−/− females that are viable and fertile. The care and use of experimental animals described in this work were approved by and comply with guidelines and policies of the University of Committee on Animal Resources at the University of Rochester.

### 4.2 Histology analysis

Samples were fixed, paraffin‐embedded, sectioned and stained with hematoxylin/eosin for histological evaluation.^11^, ^29^, ^30^, ^31^, ^32^, ^33^, ^34^ Images were taken using Leica DM2500 microscope with a DFC7000T digital imaging system (Leica Biosystems Lnc., Buffalo Grove, Illinois), as well as Nikon SMZ1500 and TS100‐F microscope (Nikon, Melville, New York) equipped with a SPOT Pursuit Slider and Insight Camera, respectively (Diagnostic Instruments, Sterling Heights, Michigan).^29^, ^30^, ^35^, ^36^, ^37^

## CONFLICT OF INTEREST

The authors declare no potential conflict of interest.
